# Cytogenomics of *Frieseomelitta varia* (Hymenoptera: Apidae) and the Sharing of a Satellite DNA Family in Several Neotropical Meliponini Genera

**DOI:** 10.3390/genes16010086

**Published:** 2025-01-15

**Authors:** Zulemara B. M. Vignati, Gisele A. Teixeira, Marina S. Cunha, Jaqueline A. Pereira, Denilce M. Lopes

**Affiliations:** 1 Laboratório de Citogenética de Insetos, Departamento de Biologia Geral, Universidade Federal de Viçosa, Campus Universitário, Viçosa 36570-900, Minas Gerais, Brazil; zulemara.bio@gmail.com (Z.B.M.V.); g.amaroteixeira@gmail.com (G.A.T.); marina.souza.cunha@gmail.com (M.S.C.); jaquelineamorimn@gmail.com (J.A.P.); 2 Instituto de Veterinária, Universidade Federal Rural do Rio de Janeiro, Campus Seropédica, Seropédica 23891-970, Rio de Janeiro, Brazil

**Keywords:** evolution, genome, heterochromatin, repetitive DNA, stingless bees

## Abstract

Background/Objectives: A striking feature of the karyotypes of stingless bees is the large amount of heterochromatin present in most species. Cytogenomic studies performed in some Meliponini species have suggested that evolutionary events related to the diversification and amplification of satellite DNA families in the heterochromatin may reflect the structuring of phylogenetic clades in this tribe. In this study, we performed a genomic analysis in *Frieseomelitta varia* to characterize different satDNA families in its genome. We also investigated the presence of the most abundant satDNA family of *F. varia* in its own chromosomes, in two other *Frieseomelitta* species, and in other Meliponini genera encompassing the three main clades of Neotropical Meliponini, according to the available molecular phylogeny. Methods: Genomic analyses were performed using RepeatExplorer2 on the Galaxy platform, and chromosomal investigations were conducted using fluorescent in situ hybridization. Results: Seven satDNA families were recovered, which together totaled an abundance of 11.223% of the analyzed *F. varia* genomic fraction. The most abundant satDNA family, FvarSat01-306, predominates in the analyzed repetitive fraction (representing around 89%) and was recently amplified and homogenized in almost all the heterochromatin of *F. varia*. In addition, the data revealed an unprecedented sharing of this satDNA family in the centromeric/pericentromeric heterochromatin among different Meliponini genera, with independent amplifications and loss of this sequence in some taxa. Conclusions: One family of satellite DNA makes up most of the heterochromatin in this species and is shared with other Meliponini.

## 1. Introduction

The constitutive heterochromatin (*c*-heterochromatin) domain is a chromosomal region that remains highly condensed throughout the cell cycle and is typically enriched with repetitive sequences such as satellite DNA (here called satDNA) and transposable elements [1,2]. This type of chromatin plays an important role in the assembly of CenH3 (centromere-specific histone H3 protein) into nucleosomes and kinetochores, influencing correct chromosome segregation and maintaining genomic stability by restricting the activity of mobile elements and preventing the non-homologous recombination of repetitive sequences during DNA repair [2,3,4].

As a result of the classic cytogenetic studies conducted on stingless bees (Meliponini tribe), the importance of heterochromatin in the chromosomal evolution of these insects has already been discussed. Most species were found to have a significant amount of heterochromatin located along one of the chromosomal arms or even throughout almost the entire chromosomal length [5]. Consistent with these findings, amplifications of heterochromatin have been suggested as the origin of genomes with higher 1C values (the amount of DNA in the haploid nuclear genome) observed in some species of Meliponini, highlighting the role of heterochromatin in the genomic evolution of these bees [5,6].

Over the last few years, molecular cytogenetic studies using various methodologies for the production of specific probes (such as chromosomal microdissection, restriction enzymes, and C_0_*t*-DNA renaturation kinetics) and fluorescent in situ hybridization (FISH) in different stingless bees genera have provided the first insights into the composition and evolution of heterochromatin in Meliponini [7,8,9,10]. These studies revealed that heterochromatin contains a highly abundant repetitive DNA sequence that is shared between chromosomes of the same species and between closely related species of the same genus but not between the different genera. However, a recent study performed by Campos et al. [11] using C_0_*t*-DNA has revealed the sharing of repetitive sequences between the genera *Plebeia* and *Friesella* in part of the heterochromatin.

However, the identity of the sequences constituting heterochromatin remained unknown until recently. With the advent of cytogenomic approaches—which integrate genomic information, chromosomal data, and bioinformatics tools—a deeper understanding of the composition and evolution of heterochromatin in Meliponini has begun to emerge [12,13]. The first study using this approach in stingless bees was conducted in the genus *Melipona*, showing that despite sharing several satDNA families between species, the most abundant satDNA is unique within the subgenera *Michmelia* and *Melipona,* which have very disparate heterochromatin patterns, i.e., high and low heterochromatin content, respectively [12]. In addition, although the satellitome (the set of satDNAs in the genome) of these species shows homology in eleven satDNA families, these sequences have undergone different amplification and retraction processes over time [12]. These findings are consistent with the library hypothesis, which proposes that a group of related species may share satDNA families inherited from a common ancestor, but these families can evolve differently in each species [14].

Similar results were observed with the characterization of the stingless bee *Trigona hyalinata* satellitome [13]. The authors demonstrated that *T. hyalinata* heterochromatin is also composed of a predominant satDNA (ThyaSat01-301) that is highly abundant in this region. By associating phylogenetic and cytogenomic data [13,15], the reults showed that this satDNA is shared with other *Trigona* species of the same clade but not observed in species of the sister clade, indicating a divergent evolution of heterochromatin in the two clades of *Trigona*. Thus, cytogenomic studies on *Melipona* and *Trigona* have suggested that evolutionary events related to the diversification and amplification of the satellitome may reflect the structuring of phylogenetic clades in the Meliponini tribe [12,13].

Recently, genome sequencing data from the stingless bee *F. varia* became available, revealing a large proportion of repetitive components [16], presenting an opportunity to use this data in cytogenomic analyses. Cytogenetic studies on *F. varia* show that this species has 2n = 30 chromosomes, with heterochromatin distributed in the centromeric region of the chromosomes and along one arm in most pairs [5,16,17]. Additionally, 12 other taxa of *Frieseomelitta* have been studied cytogenetically, showing a conserved chromosome number of 2n = 30, with the exception of *Frieseomelitta longipes*, with 2n = 34 [5,16,17,18,19,20]. C-banding data for some of these species show a high heterochromatic content similar to the pattern observed in *F. varia* [5,16,17,18,19].

In this study, we performed a genomic analysis in *F. varia* to identify different satDNA families in its genome and to characterize these sequences in terms of their abundance and divergence. We performed chromosomal mapping of the most abundant satDNA family of *F. varia* across three *Frieseomelitta* taxa to determine if this sequence is the main constituent of heterochromatin in these taxa and whether it is shared within the genus. Additionally, we investigated the presence of this predominant satDNA in other Meliponini genera, both phylogenetically close to and distant from *Frieseomelitta*, according to the molecular phylogeny proposed by Rasmussen and Cameron [21]. The results obtained from these experiments provided new insights into the evolution of satDNA in the Meliponini tribe.

## 2. Materials and Methods

### 2.1. Analysis of satDNA in the Genome of the Stingless Bee F. varia

The raw genome data of *F. varia* were obtained from the publicly accessible database Sequence Read Archive (SRA)-NCBI, with the accession number SRX6799462. The authors used NGS on the Illumina HiSeq2500 platform to generate a library of short paired-end reads (2 × 101 bp) [16]. The quality of the genomic data was inspected using the FastQC Read Quality Reports tool, implemented on the public platform Galaxy (https://repeatexplorer-elixir.cerit-sc.cz/galaxy, accessed on 1 February 2022). The satDNAs from the genome of *F. varia* were identified using RepeatExplorer2 (Galaxy Version 2.3.8.1), available on the Galaxy platform (accessed on 3 February 2022). These analyses followed the protocol of Novák et al. [22]. The pre-processing of the paired-end reads was performed, which included trimming, quality filtering, Cutadapt filtering, and interlacing of genomic reads. RepeatExplorer2 clustering was performed with default parameters using a random sample of one million reads. After this, the identification of satDNAs was performed using the TAREAN tool [23]. We compared the identified satDNA sequences to check a possible common identity between them using the Geneious software V4.8 [24]. Considering their level of sequence identity, satDNA sequences were classified according to the methods of Ruiz-Ruano et al. [25] into families (>80%) and superfamilies (>60%). In addition, the nomenclature of the identified satDNAs was performed according to the methods of Ruiz-Ruano et al. [25]. These sequences were deposited in GenBank with the following accession numbers: PQ390412, PQ390413, PQ390414, PQ390415, PQ390416, PQ390417, and PQ390418.

The abundance and divergence of each satDNA family was estimated by means of RepeatMasker [26] using the Cross_match. A total of ten million randomly selected reads were used for this analysis. The abundance of each satDNA family was estimated based on its proportion in the genome, and the sum of the mapped nucleotides belonging to one specific satDNA was divided by the total number of nucleotides in the library. The divergence of the satDNAs was determined using the Kimura two-parameter (K2P) model using the alcDivergenceFromAlign.pl script in RepeatMasker software v4.1.4 [26]. The repeat landscapes were generated by comparing the sequence divergence versus the abundance of each satDNA family.

### 2.2. Obtaining Metaphase Chromosomes and Heterochromatin Distribution Patterns in Meliponini Species

Mitotic chromosomes from different stingless bee species (Table 1) were obtained from the cerebral ganglia of larvae after meconium elimination, following the protocol described by Imai et al. [27]. In total, 17 species were analyzed, covering the three clades of Neotropical Meliponini: *Trigonisca* clade (Clade 1), *Melipona* clade (Clade 2), and the remaining clade that includes the other Neotropical genera (Clade 3), according to the molecular phylogeny proposed by Rasmussen and Cameron [21].

To determine heterochromatin distribution patterns, the C-banding technique was performed according to the methods of Sumner [28], with adaptations regarding incubation times in specific solutions: 1 min in HCl, 6 min in Ba(OH)_2_, and 5 min in 2 × SSC for *Frieseomelitta* species; 1 min in HCl, 4 min in Ba(OH)_2_, and 4 min in 2 × SSC for *Duckeola ghilianii*; and 1 min in HCl, 6 min Ba(OH)_2_, and 6 min in 2 × SSC for *Partamona helleri*. In addition, sometimes, conventional staining with 4% Giemsa, without any pretreatment with hydrochloric acid, barium hydroxide, and saline, is sufficient to observe heterochromatin distribution patterns in chromosomes [27,29], which was the case for the other stingless bee species (Table 1).

### 2.3. Chromosomal Mapping of the Most Abundant satDNA Family from the F. varia Genome Using Fluorescent In Situ Hybridization (FISH)

In order to investigate the presence of the most abundant satDNA family (FvarSat01-306) of the *F. varia* genome in the karyotypes of other species of *Frieseomelitta* and other Meliponini genera (Table 1), we produced a probe of this satDNA via polymerase chain reaction (PCR) using the following manually designed specific primers: F- 5′ATTTCGTGTTTCTCGATGCCA 3′ and R- 5′ATTTCGGCTAGATTCGAAGATA 3′. The genomic DNA of *F. varia* was extracted using the DNeasy Kit (Qiagen Inc., Redwood City, CA, USA), following the manufacturer’s instructions. The PCR solutions totaling 10 μL contained 5.52 μL of H_2_O, 1 μL of template DNA (100 ng/μL), 0.4 μL of each primer (10 mmol/L) (forward and reverse), 2 μL of 5 × Color-less GoTaq Flexi buffer, 0.08 μL of dNTPs (20 mmol/L), 0.4 μL of MgCl_2_ (25 mmol/L), and 0.2 μL of GoTaq Flexi DNA Polymerase (5 U/μL) (Promega, Maddison, WI, USA). The PCR conditions included an initial denaturation step at 94 °C for 5 min and 30 cycles at 94 °C (30 s), 60 °C (30 s), and 72 °C (80 s), plus a final extension at 72 °C for 5 min. The PCR products were checked using a 1% agarose gel.

Mapping of the FvarSat01-306 probe was performed using the FISH technique, according to the methods of Pinkel et al. [30]. The FvarSat01 probe was labeled using the indirect method with digoxigenin-11-dUTP using the Dig-Nick-Translation Mix kit (Roche Applied Science, Mannheim, Germany). The identification of probe signals was performed using anti-digoxigenin-rhodamine (Roche Applied Science, Mannheim, Germany). The chromosomes were counterstained with 4′,6-Diamidino-2-Phenylindole (DAPI) (Sigma-Aldrich, Darmstadt, Germany). The metaphases were captured on an Olympus BX53F microscope equipped with an Olympus XM10 camera using CellSens imaging software v1.12 and WG (510–550 nm) filters for rhodamine and WU (330–385 nm) filters for DAPI. At least 30 metaphases per species were used to determine the patterns, employing the FISH technique. The karyotypes were organized using Adobe Photoshop^®^ CS6 software.

Additionally, we performed a parallel analysis to investigate the presence of the most abundant satDNA family from the *T. hyalinata* genome (ThyaSat01-301) in the chromosomes of closely related genera, including *Cephalotrigona*, *Scaptotrigona*, *Tetragona*, and *Geotrigona.* These species form a branch within Clade 3, according to the molecular phylogeny proposed by Rasmussen and Cameron [21]. The satDNA ThyaSat01-301 probe was produced using specific primers designed by Pereira et al. [13] and template DNA from *T. hyalinata*. The preparation of this probe, as well as the FISH assays, followed the methodologies described previously.

## 3. Results

### 3.1. Genomic Analysis of satDNAs in F. varia

Bioinformatic analyses identified seven satDNA families, which together totaled an abundance of 11.223% of the *F. varia* genome, considering the genomic fraction analyzed (Table 2). The most abundant satDNA family (FvarSat01-306) represented 9.971% of the genome, while the other satDNAs together corresponded to only 1.252%. Therefore, the FvariaSat01-306 satDNA is approximately eight times more abundant in the *F. varia* genome compared to all other satDNA families combined. The lowest abundance was observed in the satDNA family FvariaSat07-145, with 0.014% (Table 2).

The length of the monomers varied from 131 to 1445 bp. The three most abundant satDNA families, namely FvarSat01, FvarSat02, and FvarSat03, possessed the largest monomers, with 306 bp, 449 bp, and 1145 bp, respectively. The A + T base pair content of the satDNA families ranged from 53.2 to 72.5%, with an average of 62.15% (Table 2). These values show a predominance of A+T base pairs in the different satDNA families identified, which follows the whole genome pattern of *F. varia*, with an estimated A+T base content of 63%, see [16]. Comparative analysis between the satDNA sequences showed a homology greater than 50% between two families (FvarSat04-145 and FvarSat06-145), allowing them to be grouped into the same superfamily (SF1).

**Figure 1:**
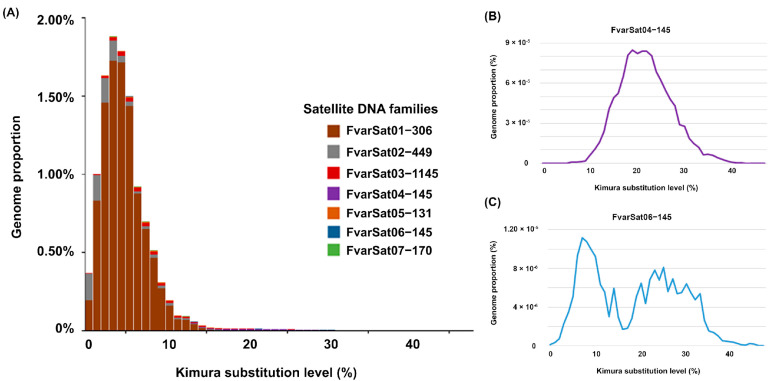
Landscape (abundance versus divergence) for the satDNA families identified in the *F. varia* genome (**A**). Note in (**A**) the predominance of the FvarSat01-306 family in relation to the other satDNAs. This family showed an abundance peak at low divergence values (about 5%), revealing its recent amplification and high sequence homogenization. Landscapes in (**B**,**C**) for the FvarSat04-145 and FvarSat06-145 families, respectively, show an abundance peak at high divergence values, indicating the accumulation of mutations in these sequences.

The divergence between satDNA families ranged from 2.9% to 22.09%, with an average of 10.07% (Table 2). According to the landscape graph (abundance versus divergence), five satDNA families, including the most abundant in the *F. varia* genome, showed an abundance peak at low divergence (around 5%), indicating recent amplifications of these families with high sequence homogenization (Figure 1A and Figure S1). However, the satDNA families FvarSat04-145 and FvarSat06-145, which are included in the same superfamily, showed an abundance peak at higher divergence values, indicating the accumulation of mutations in these sequences (Figure 1B,C and Figure S1).

### 3.2. Chromosomal Analysis: C-Banding and Physical Mapping of the Most Abundant satDNA Family (FvarSat01-306) in Different Meliponini Species

**Figure 2:**
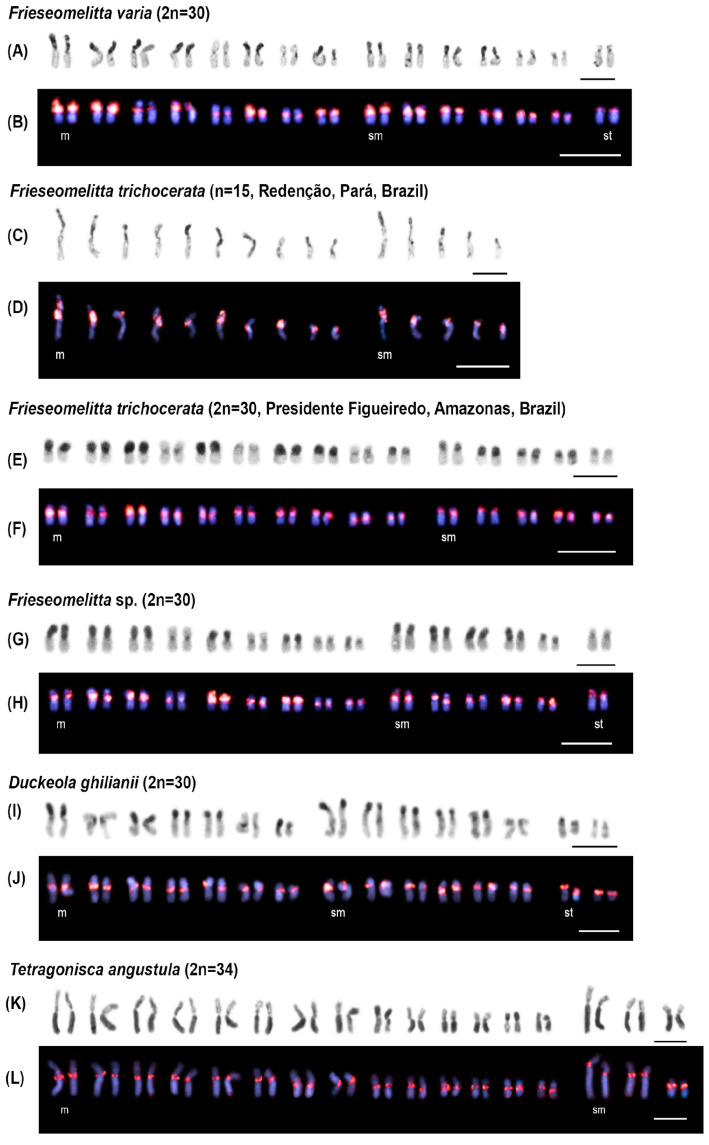
Karyotypes of five stingless bee species showing heterochromatin distribution patterns (darker regions) in (**A**,**C**,**E**,**G**,**I**,**K**). Karyotypes subjected to fluorescent in situ hybridization with the most abundant satDNA family probe FvarSat01-306 (red regions) and counterstaining with DAPI (blue regions) in (**B**,**D**,**F**,**H**,**J**,**L**). Chromosomal classification: m = metacentric, sm = submetacentric, and st = subtelocentric. Bars = 5 µm.

**Figure 3:**
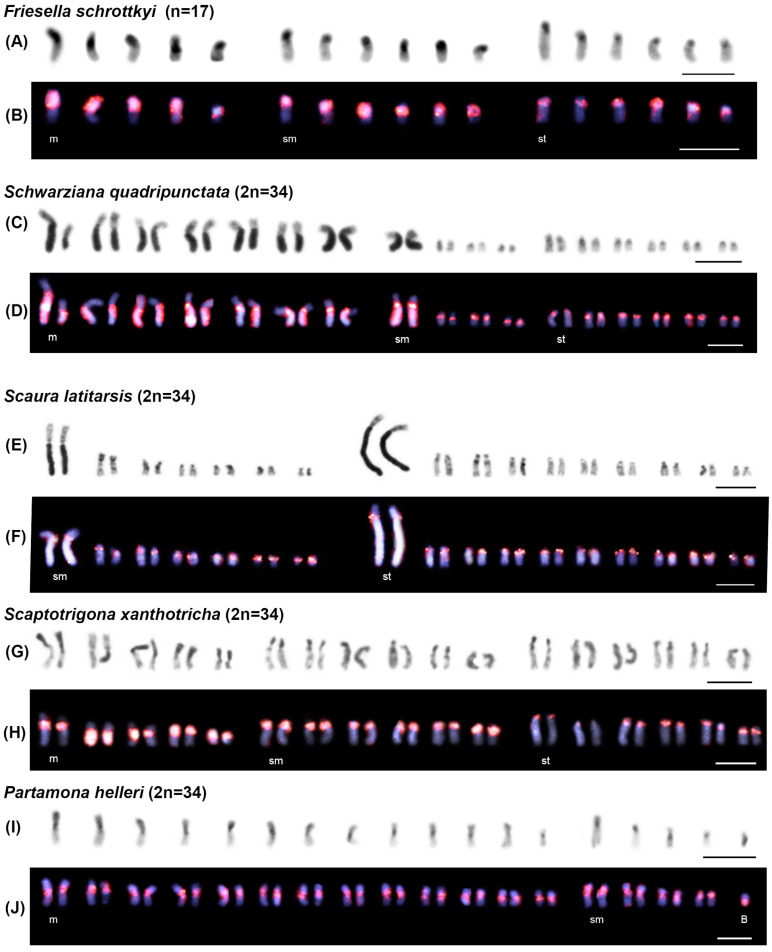
Karyotypes of five stingless bee species showing heterochromatin distribution patterns (darker regions) in (**A**,**C**,**E**,**G**,**I**). Karyotypes subjected to fluorescent in situ hybridization with the most abundant satDNA family probe FvarSat01-306 (red regions) and counterstaining with DAPI (blue regions) in (**B**,**D**,**F**,**H**,**J**). Chromosomal classification: m = metacentric, sm = submetacentric, and st = subtelocentric. The letter “B” in *P. helleri* karyotypes indicates the B chromosome. Bars = 5 µm.

In general, the distribution of heterochromatin observed in the studied species was predominantly located at the centromeres and along one chromosome arm (either short or long) in most or all chromosomes (Figure 2 and Figure 3). However, a few chromosome pairs exhibited heterochromatin only in the centromeric region, as seen in *F. varia* (pairs 5th m, 7th m, 6th sm, and 1st st) (Figure 2A), *Frieseomelitta trichocerata* (pairs 4th m, 6th m, and 9th m) (Figure 2C,E), *Frieseomelitta* sp. (pairs 4th m, 6th m, 8th m, and 1st st) (Figure 2G), and *D. ghilianii* (pairs 2nd m, 6th m, and 6th sm) (Figure 2I). Additionally, some specific patterns in heterochromatin distribution were observed in *Schwarziana quadripunctata*, *Scaura latitarsis*, and *P. helleri*. The former species displayed eight larger chromosome pairs (1 to 8) with very long heterochromatic arms and nine smaller pairs with short heterochromatic arms (Figure 3C). The second species showed two larger pairs (1 and 8) with very long heterochromatic arms, while the rest of the chromosomes were smaller with short heterochromatic arms (Figure 3E). The last species exhibited two heterochromatic regions with varying intensities: the centromere showed darker staining, while the short arms of some chromosomes displayed lighter staining. Additionally, most of the B chromosome observed in this species was heterochromatic, except for one end, which was euchromatic (Figure 3I).

FISH assays with the FvarSat01-306 probe revealed different hybridization patterns among the analyzed stingless bee species (Figure 2 and Figure 3). This satDNA family was present in almost all the heterochromatin of *F. varia* (Figure 2B) and in the centromeric/pericentromeric heterochromatin of all chromosomes, as well as in the short arm of only one pair in *F. trichocerata* (Figure 2D,F) and *Frieseomelitta* sp. (Figure 2H). The probe showed positive signals exclusively in the centromeric heterochromatin of *D. ghilianii* (Figure 2J), *Tetragonisca angustula* (Figure 2L), and *S. latitarsis* (Figure 3F), while marking all the heterochromatin in *Friesella schrottkyi* (Figure 3B) and *Scaptotrigona xanthotricha* (Figure 3H). In *S. quadripunctata*, FvarSat01-306 was present at the centromeres of all chromosomes and along the long heterochromatic arms of the eight largest pairs (Figure 3D). Lastly, *P. helleri* showed positive signals in the centromeric/pericentromeric heterochromatin of all chromosomes and in most of the heterochromatin of the B chromosome (Figure 3J).

No hybridization signal with the FvarSat01-306 probe was observed in the chromosomes of *Geotrigona subterranea*, *Tetragona elongata*, *Trigona spinipes*, or *Cephalotrigona capitata*, which also belong to Clade 3, nor in *Leurotrigona muelleri*, *Melipona quadrifasciata*, or *Melipona mondury* (Figure S2), which are included in the other two Neotropical clades. In addition, no hybridization signal with the satDNA family ThyaSat01-301 probe from the *Trigona hyalinata* genome was observed in the chromosomes of *G. subterranea*, *T. elongata*, *C. capitata*, and *S. xanthotricha* (Figure S3).

**Figure 4:**
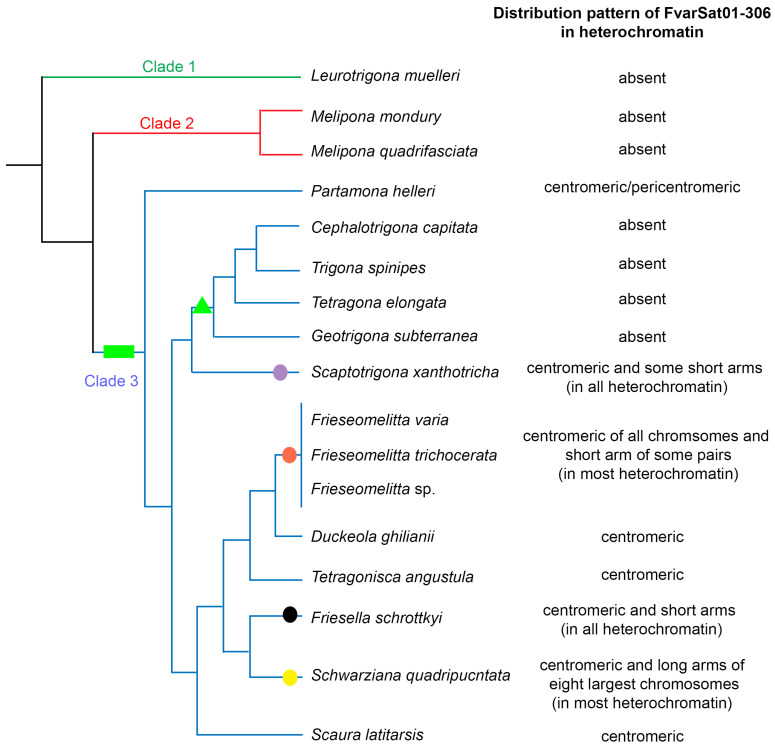
Data on the chromosomal location of the most abundant satDNA family FvarSat01-306 in the heterochromatin of the analyzed stingless bees associated with the phylogenetic relationships of these species, according to Rasmussen and Cameron (2010). Symbols represent hypotheses about evolutionary events related to the satDNA FvarSat01-306 across Clade 3 species: the green rectangle indicates the presence of this family in the ancestor of Clade 3; the green triangle signifies the loss of this family in the ancestor of the branch that includes genera *Geotrigona*, *Tetragona*, *Trigona*, and *Cephalotrigona*; the purple, orange, black, and yellow circles represents independent amplifications of this family in the heterochromatin of distinct genera.

Figure 4 summarizes the data on the chromosomal location of the most abundant satDNA family FvarSat01-306 in the heterochromatin of the analyzed stingless bees associated with the phylogenetic relationships of these species, according to Rasmussen and Cameron [21].

## 4. Discussion

Bioinformatic analysis recovered seven satDNA families in the *F. varia* genome that varied in abundance. The satDNA family FvarSat01-306 constituted 88.8% of this class of repetitive DNA in the analyzed genomic fraction of *F. varia*. FISH analysis showed that satDNA family FvarSat01-306 comprises almost the entire heterochromatin of *F. varia* located in centromeric/pericentromeric regions of all the chromosomes and the short arms of some pairs. In addition, this satDNA family showed a high abundance in low divergence values, indicating that it underwent a recent amplification process and sequence homogenization [25,31] in the heterochromatin of *F. varia*. Similar results were observed in the stingless bees of the *Melipona* and *Trigona* genera, which have a significant amount of heterochromatin. Their satellitomes were predominantly composed of a specific satDNA family that was recently amplified and homogenized in the heterochromatin, becoming the main constituent of this chromosome region [12,13]. This pattern may represent a trend in Meliponini, and the increase in studied species through cytogenomics may confirm this hypothesis.

Our FISH experiments also showed that this satDNA family is shared with other species of the same genus, i.e., *F. trichocerata* and *Frieseomelitta* sp., indicating an amplification event in *Frieseomelitta*. However, in these two species, the distribution of this satDNA was somewhat more restricted, indicating variations between these species that may reflect phylogenetic structures within the genus. As observed in *Melipona* species, other satDNA families and/or transposable elements may constitute the remainder of the heterochromatin [12]. A robust phylogeny for *Frieseomelitta*, along with the cytogenomic analysis of other species, may clarify these questions.

The above-mentioned species belong to Clade 3 in the molecular phylogenetic analyses of the Meliponini tribe [21]. Therefore, we also investigated the presence of satDNA family FvarSat01-306 in different representatives of the branches that comprise this clade, revealing an unprecedented sharing of this satDNA among different genera and an independent amplification and loss of this sequence in some taxa (see Figure 4). The most frequent pattern was the presence of this satDNA family exclusively in the centromeric heterochromatin (i.e., *Partamona*, *Scaura*, *Nannotrigona*, *Tetragonisca*, and *Duckeola*), indicating that, in these genera, a different satDNA family constitutes the main portion of the heterochromatin. However, this satDNA family was independently amplified to compose the main heterochromatic sequence in *Schwarziana*, *Friesella*, and *Scaptotrigona*. Interestingly, the other genera that comprise the branch to which *Scaptotrigona* belongs, i.e., *Cephalotrigona*, *Trigona*, *Tetragona*, and *Geotrigona*, lost the satDNA FvarSat01-306.

The satDNA FvarSat01-306 must have appeared in the ancestral representative of Clade 3 around 44 MYA [21], since it is not present in the species of Clades 1 and 2, i.e., *Leurotrigona* and *Melipona*, respectively (Figure 4). After the diversification of Clade 3, this satDNA evolved independently by differential contractions and amplification processes, as predicted by the library hypothesis [14]. Although satDNA can be shared between species, its abundance changes stochastically through expansion and reduction, resulting in interspecifically distinct profiles, which are even observed between lineages/populations within a species [31,32,33,34,35,36,37,38,39]. Recently, Camacho et al. [31] discussed a model for the evolution of satDNAs, suggesting that satellites undergo repeated amplification–degeneration cycles that can maintain them in the genome for a long period of time through unequal crossing-over or can lead to their disappearance by sequence degeneration through point mutations.

Alternatively, Fry and Salser [40] proposed that the acquisition of a biological function is a determining factor for a satDNA to be maintained over time in the library during long evolutionary periods. The presence of satDNA family FvarSat01-306 in the centromeric heterochromatin of species for at least 44 MYA leads us to speculate regarding whether or not this sequence possesses a possible function that would cause it to be maintained by natural selection for such a long period of time. SatDNAs are known to be involved in functional interactions necessary for centromere stability and evolution (reviewed in [41]).

In *Trigona*, which is subdivided into two clades that diversified around 19 MYA [15], cytogenomic analysis showed that the satDNA ThyaSat01-301 is shared by species of the same clade, but no signals were found in species belonging to the sister clade [13]. Since *Trigona*, *Cephalotrigona*, *Tetragona*, and *Geotrigona* did not show any signals of FvarSat01-306 in their chromosomes, we tested to determine whether satDNA ThyaSat01-301 (from *Trigona hyalinata*) could be the sequence amplified in this branch of species. This satellite probe hybridized only in *Trigona* chromosomes, showing no signals in *Cephalotrigona*, *Tetragona*, *Geotrigona*, nor in *Scaptotrigona*, indicating diversification in the composition of heterochromatin in different Meliponini branches. Future studies should identify the main constituent of heterochromatin in these species.

The present study showed the evolution of one satDNA family with an ancient origin in Clade 3 of Neotropical Meliponini, with stochastic amplification and loss in different taxa. The available body of data point to the important role of satDNA in the evolution of heterochromatin in this group of bees and its possible role in species diversification. These results reinforce the relevance of further efforts to elucidate and understand the mechanisms that lead to the evolution of these sequences, as well as their biological role.

## Figures and Tables

**Table 1:** Stingless bee species (Meliponini) analyzed in the cytogenetic experiments. Species are distributed in the three main clades (Clade 1: *Trigonisca*, Clade 2: *Melipona*, and Clade 3: most Neotropical Meliponini genera), according to the phylogeny proposed by Rasmussen and Cameron [21], and their respective geographic locations: Brazilian states, MG—Minas Gerais, AM—Amazonas, PA—Pará, and DF—Distrito Federal.

Species	Phylogenetic Clades	Locality
*Frieseomelitta varia* Lepeletier, 1836	Clade 3	Uberlândia, MG, Brazil
*Frieseomelitta trichocerata* Moure, 1990	Clade 3	Redenção, PA, Brazil; Presidente Figueiredo, AM, Brazil
*Frieseomelitta* sp.	Clade 3	Brasília, DF, Brazil
*Duckeola ghilianii* Spinola, 1853	Clade 3	Presidente Figueiredo, AM, Brazil
*Tetragonisca angustula* Latreille, 1811	Clade 3	Mutum, MG, Brazil
*Friesella schrottkyi* Friese, 1900	Clade 3	Rio Paranaíba, MG, Brazil
*Schwarziana quadripunctata* Latreille, 1836	Clade 3	Viçosa, MG, Brazil
*Scaura latitarsis* Friese, 1900	Clade 3	Presidente Figueiredo, AM, Brazil
*Scaptotrigona xanthotricha* Moure, 1950	Clade 3	Viçosa, MG, Brazil
*Geotrigona subterranea* Friese, 1901	Clade 3	Passos, MG, Brazil
*Tetragona elongata* Lepeletier & Serville, 1828	Clade 3	Viçosa, MG, Brazil
*Trigona spinipes* Fabricius, 1793	Clade 3	Januária, MG, Brazil
*Cephalotrigona capitata* Smith, 1854	Clade 3	Viçosa, MG, Brazil
*Partamona helleri* Friese, 1900	Clade 3	Viçosa, MG, Brazil
*Melipona quadrifasciata* Lepeletier, 1836	Clade 2	Viçosa, MG, Brazil
*Melipona mondury* Smith, 1863	Clade 2	Viçosa, MG, Brazil
*Leurotrigona muelleri* Friese, 1900	Clade 1	Passos, MG, Brazil

**Table 2:** Main characteristics of satellite DNA (satDNA) families identified in the genome of *F. varia*.

SatDNA Family	SF	ML	A + T%	Divergence %	Abundance %
FvarSat01-306		306	66	4.62	9.971%
FvarSat02-449		449	53.2	2.92	0.803%
FvarSat03-1145		1145	58.4	7.73	0.245%
FvarSat04-145	1	145	54.5	22.09	0.117%
FvarSat05-131		131	72.5	8.67	0.054%
FvarSat06-145	1	145	59.3	18.36	0.019%
FvarSat07-170		170	71.2	6.13	0.014%
Total					11.223%

SF = superfamilies; ML = monomer length; A + T% = content A + T.

## Data Availability

The original contributions presented in this study are included in the article and Supplementary Material. Further inquiries can be directed to the corresponding author.

## Supplementary Materials

The following supporting information can be downloaded at: https://www.mdpi.com/article/10.3390/genes16010086/s1, Figure S1. Landscapes (abundance versus divergence) for each satellite DNA family identified on the genome of *Frieseomelitta varia*; Figure S2. Fluorescent in situ hybridization with the most abundant satDNA family probe FvarSa01-306 in different stingless bee species: (A) *Geotrigona subterranea*, (B) *Tetragona elongata*, (C) *Cephalotrigona capitata*, (D) *Trigona spinipes*, (E) *Leurotrigona muelleri*, (F) *Melipona quadrifasciata*, and (G) *Melipona mondury*; Figure S3. Fluorescent in situ hybridization with the most abundant satDNA family probe (ThyaSat01-301) from *Trigona hyalinata* genome in different stingless bee species: (A) *Geotrigona subterranea*, (B) *Tetragona elongata*, (C) *Cephalotrigona capitata*, and (D) *Scaptotrigona xanthotricha*. The probe did not hybridize to any region of the chromosomes in these species. Chromosomes were counterstained with DAPI. Bars = 5 µm. The probe did not hybridize to any region of the chromosomes in these species. Chromosomes were counterstained with DAPI. Bars = 5 µm.
