# Supplementary material for: Cytogenomics of Frieseomelitta varia (Hymenoptera: Apidae) and the Sharing of a Satellite DNA Family in Several Neotropical Meliponini Genera
Source: Genes (Basel). 2025 Jan 15;16(1):86. doi: 10.3390/genes16010086 (PMC11764717; doi:10.3390/genes16010086)
Supplement: Supplementary file 1 [file genes-16-00086-s001.zip › genes-3372388-supplementary.pdf]

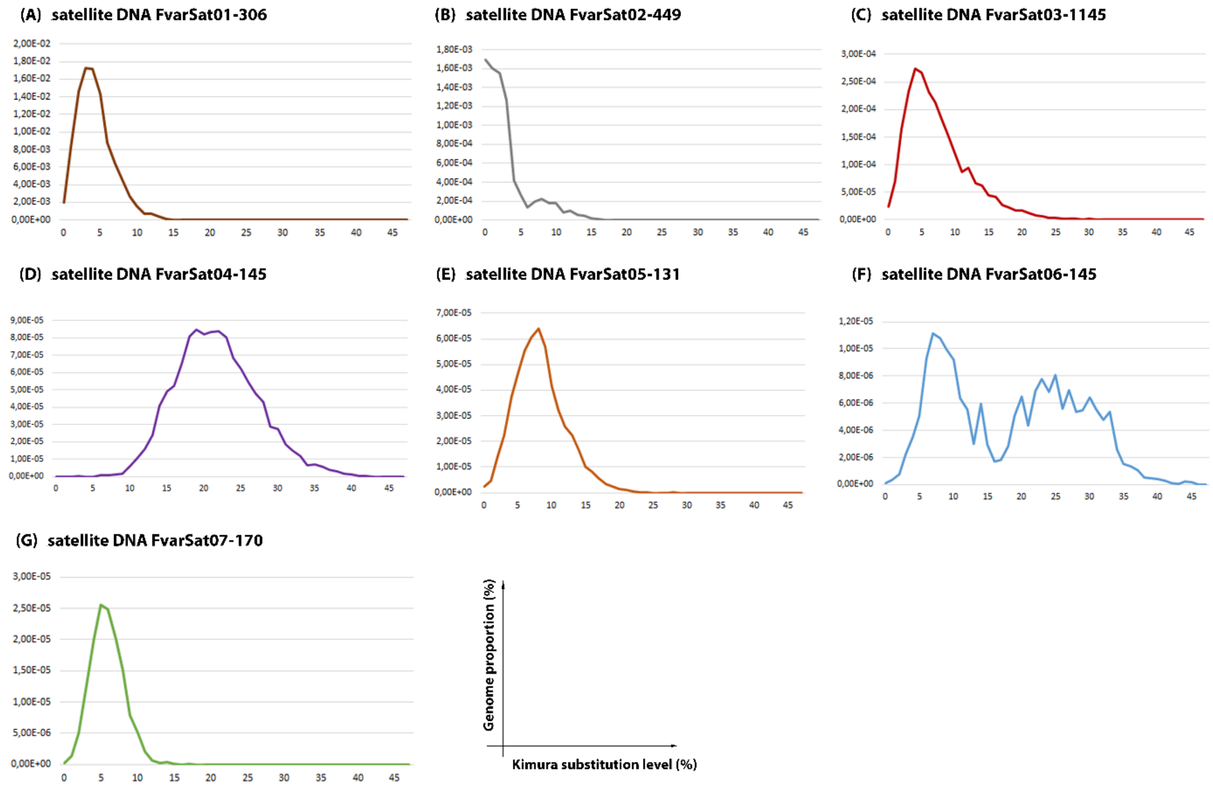

Figure S1. Landscapes (abundance versus divergence) for each satellite DNA family identified on the genome of *Frieseomelitta varia*.

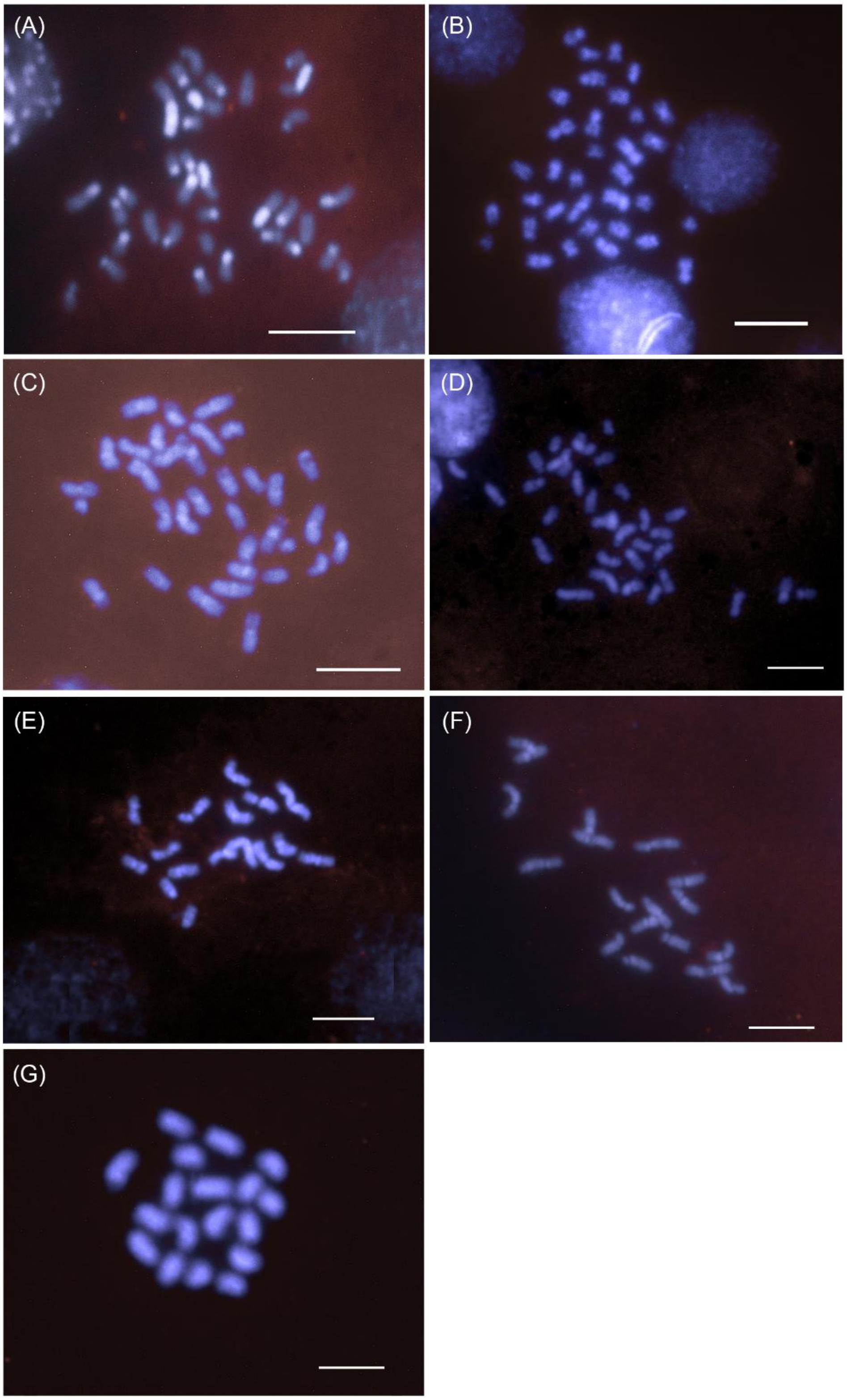

Figure S2. Fluorescent *in situ* hybridization with the most abundant satDNA family probe FvarSa01-306 in different stingless bee species: (A) *Geotrigona subterranea*, (B) *Tetragona elongata*, (C) *Cephalotrigona capitata*, (D) *Trigona spinipes*, (E) *Leurotrigona muelleri*, (F) *Melipona quadrifasciata*, and (G) *Melipona mondury*. The probe did not hybridize to any region of the chromosomes in these species. Chromosomes were counterstained with DAPI. Bars = 5 µm.

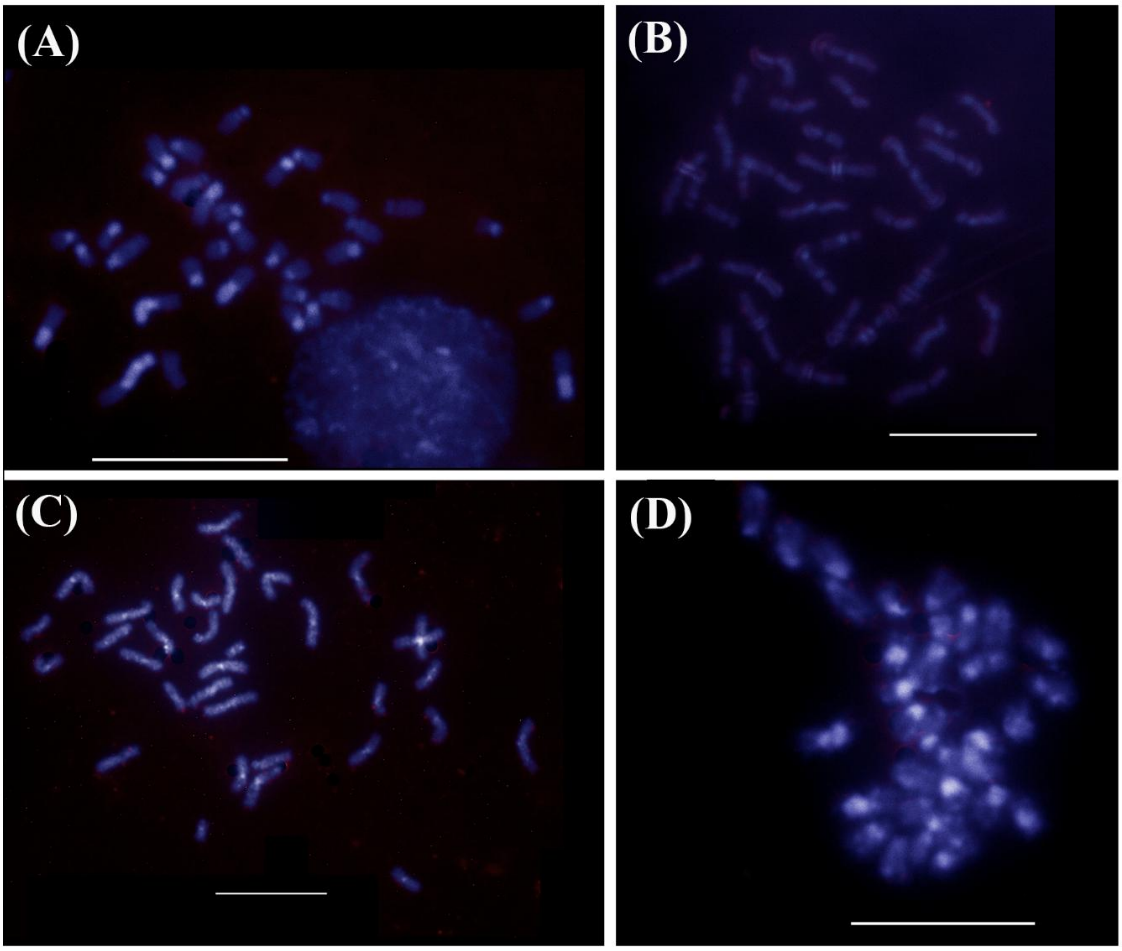

Figure S3. Fluorescent *in situ* hybridization with the most abundant satDNA family probe (ThyaSat01-301) from *Trigona hyalinata* genome in different stingless bee species: (A) *Geotrigona subterranea*, (B) *Tetragona elongata*, (C) *Cephalotrigona capitata*, and (D) *Scaptotrigona xanthotricha*. The probe did not hybridize to any region of the chromosomes in these species. Chromosomes were counterstained with DAPI. Bars = 5 μm.
